# Bis(2-nitro­phen­yl)methane

**DOI:** 10.1107/S1600536814015438

**Published:** 2014-07-05

**Authors:** Daron E. Janzen, Laura E. Crepeau, Benjamin D. Hageseth, James W. Wollack

**Affiliations:** a Department of Chemistry and Biochemistry, St Catherine University, 2004 Randolph Avenue, St Paul, MN 55105, USA

**Keywords:** crystal structure

## Abstract

In the title compound, C_13_H_10_N_2_O_4_, the nitro groups are twisted significantly relative to the benzene rings [dihedral angles = 16.64 (18) and 28.02 (11)°]. The benzene groups are nearly perpendicular to each other [dihedral angle = 87.72 (6)°]. Short inter­molecular N⋯O and C⋯O [2.981 (2) and 3.060 (2) Å, respectively] contacts suggest possible weak π-inter­actions between nitro groups and between benzene and nitro groups. In addition, there are π–π inter­actions between one benzene group and an inversion-related equivalent [inter­planar separation = 3.494 (2) Å].

## Related literature   

The synthesis of the title compound has been previously reported (Allinger & Youngdale, 1962 ▶), although by different methods from the preparation of the sample used for this study [a modification of the method given by Lu *et al.* (2006 ▶)]. For related structures, see: Barnes *et al.* (1981 ▶); Brito *et al.* (2007 ▶); Cousson *et al.* (1993 ▶); Housty (1961 ▶).
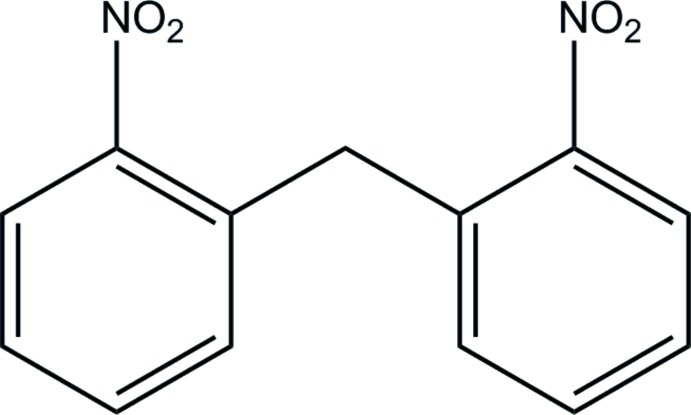


## Experimental   

### 

#### Crystal data  

C_13_H_10_N_2_O_4_
*M* *_r_* = 258.23
Triclinic, 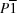

*a* = 7.628 (3) Å
*b* = 8.340 (3) Å
*c* = 9.464 (4) Å
α = 103.544 (8)°
β = 92.555 (7)°
γ = 94.870 (7)°
*V* = 582.0 (4) Å^3^
*Z* = 2
Mo *K*α radiation
μ = 0.11 mm^−1^
*T* = 173 K
0.17 × 0.15 × 0.10 mm

#### Data collection  

Rigaku XtaLAB mini diffractometer
Absorption correction: multi-scan (*REQAB*; Rigaku, 1998 ▶) *T*_min_ = 0.735, *T*_max_ = 0.989
6052 measured reflections
2648 independent reflections
1866 reflections with *F* ^2^ > 2σ(*F* ^2^)
*R*_int_ = 0.038

#### Refinement  

*R*[*F* ^2^ > 2σ(*F* ^2^)] = 0.048
*wR*(*F* ^2^) = 0.114
*S* = 1.02
2648 reflections
172 parameters
H-atom parameters constrained
Δρ_max_ = 0.19 e Å^−3^
Δρ_min_ = −0.23 e Å^−3^

### 

Data collection: *CrystalClear* (Rigaku, 2011 ▶); cell refinement: *CrystalClear*; data reduction: *CrystalClear*; program(s) used to solve structure: *SIR2004* (Burla *et al.*, 2005 ▶); program(s) used to refine structure: *SHELXL97* (Sheldrick, 2008 ▶); molecular graphics: *OLEX2* (Dolomanov *et al.*, 2009 ▶); software used to prepare material for publication: *CrystalStructure* (Rigaku, 2010 ▶).

## Supplementary Material

Crystal structure: contains datablock(s) General, I. DOI: 10.1107/S1600536814015438/pk2528sup1.cif

Structure factors: contains datablock(s) I. DOI: 10.1107/S1600536814015438/pk2528Isup2.hkl

Supporting information file. DOI: 10.1107/S1600536814015438/pk2528Isup3.cml

CCDC reference: 1011567

Additional supporting information:  crystallographic information; 3D view; checkCIF report

## Figures and Tables

**Fig. 1.**
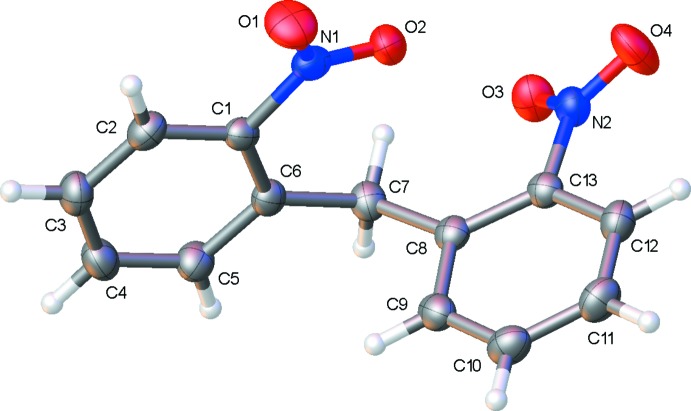
A thermal ellipsoid plot (50% probability ellipsoids for non-H atoms) of the structure of (I).

**Fig. 2.**
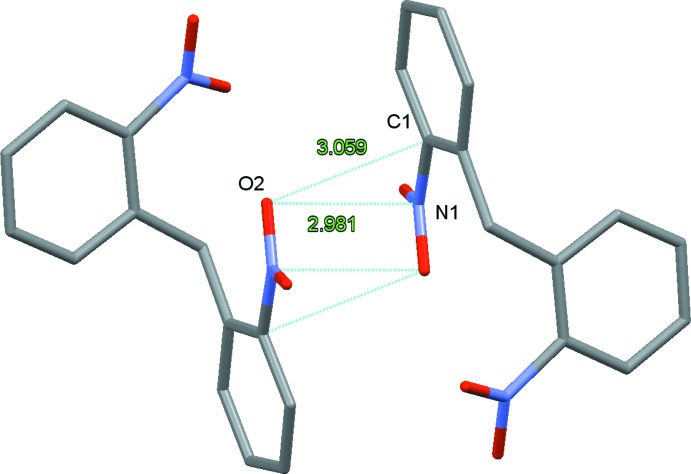
View of two molecules of (I) showing the close N1···O2 and C1···O2 contacts between two molecules related by inversion (symm. code 1 - x, 2 - y, 1 - z).

**Fig. 3.**
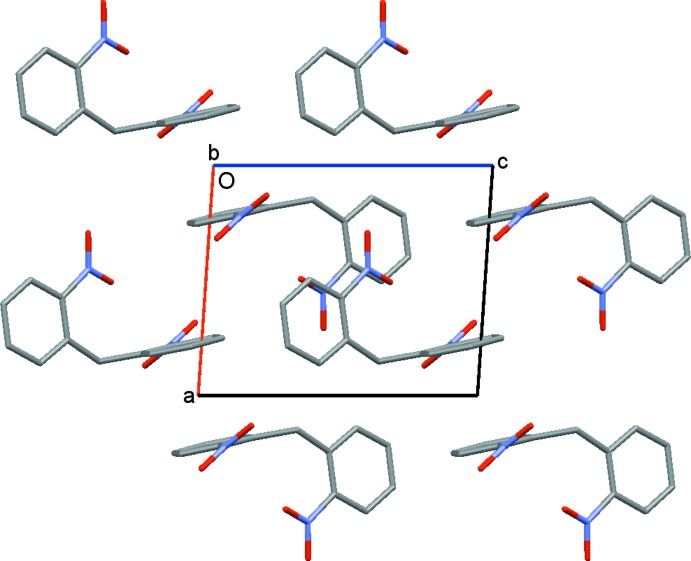
Unit cell packing diagram of (I) viewed parallel to the b axis.

## supplementary crystallographic information

### S1. Comment

4,4'-Methylene dianiline (4,4'-MDA) is principally used to produce 4,4'-methylene dianiline diisocyanate and other polymeric isocyanates, which are used to manufacture polyurethane foams. 4,4'-MDA is also used as a curing agent for epoxy resins and urethane elastomers, as a corrosion preventative for iron, as an antioxidant for lubricating oils, as a rubber processing chemical, and as an intermediate in the manufacture of elastomeric fibers. In the manufacturing process of 4,4'-MDA, by-products including 2,2'-methylene dianiline (2,2'-MDA) are produced. 2,2'-MDA can have hazardous health effects such as irritation to the skin and eyes, liver damage through acute oral or dermal exposure, and is a possible human carcinogen. In an effort to access 2,2'-MDA for use as a standard to measure the by-products created in the manufacturing process to synthesize 4,4'-MDA, we have developed a new synthesis of the intermediate 2,2'-dinitrodiphenylmethane and determined its crystal structure.

2,2'-MDA can be produced in a two-step synthesis from 2-nitrophenyl boronic acid and 2-nitrobenzyl bromide. First, 2-nitrophenyl boronic acid is reacted with 2-nitrobenzyl bromide using a Suzuki reaction to produce 2,2'-dinitrodiphenylmethane. Next, the nitro groups on the 2,2'-dinitrodiphenylmethane can be reduced using a catalytic hydrogenation reaction to produce the compound 2,2'-MDA.

The molecular structure of bis(2-nitrophenyl)methane (Fig. 1) is composed of an asymmetric unit containing one whole molecule. The N-O bond lengths (range 1.227 (2)-1.233 (2) Å) are consistent with a high degree of resonance in the nitro groups. Each nitro group is twisted from the bonded benzene moiety with angles between least-squares planes (N1, O1, O2 and C1-C6; N2, O3, O4 and C8-C13) of 16.64 (18)° and 28.02 (11)°, respectively. The benzene groups are nearly perpendicular with angles between least-squares planes of 87.72 (6)°. The orientation of the nitro groups allows for close intramolecular contacts between the oxygen atoms and methylene H atoms.

Close intermolecular contacts are also present in this structure. A short contact between N1 (*x*,*y*,*z*) and O2 (1 - *x*,2 - *y*, 1 - *z*) with a distance of 2.981 (2) Å (distance -van der Waals sum = -0.089 Å) is consistent with a weak nitro π - nitro π type interaction. These nitro groups, related by inversion, are parallel with an intermolecular distance between least-squares planes of 2.861 (3) Å. Likewise, C1 (*x*,*y*,*z*) and O2 (1-*x*, 2-*y*, 1-*z*) engage in a similar weak benzene π - nitro π type interaction at a distance of 3.060 (2) Å (distance -van der Waals sum = -0.161 Å). Short intermolecular contacts are also present between O4(*x*,*y*,*z*) ···H3(*x*,*y* + 1,*z* + 1) (2.53 Å) and O1(*x*, *y*,*z*)···H5 *x* - 1,*y*,*z*) (2.58 Å).

### S2. Experimental

Compound (I) was prepared by a modification of the method used by Lu *et al.* (2006).

Under nitrogen, a mixture of THF (5.8 ml) and aqueous K_2_CO_3_ (2*M*, 2.3 ml, 9.3 mmol) were added to 2-nitrophenylboronic acid (0.257 g, 3.08 mmol), 2-nitrobenzylbromide (0.514 g, 2.8 mmol) and Pd(PPh_3_)_4_ (0.081 g, 0.07 mmol). The reaction mixture was heated under reflux and protected from light for 24h. Aqueous HCl (1*M*, 50 ml) was added, the reaction mixture was extracted with ethyl acetate (3 *x* 20 ml), dried using MgSO_4_, and concentrated to yield a brown oil. The crude product was purified by flash chormatography (silica gel, hexanes/ethyl acetate (12:1)). Yellow X-ray quality crystals were obtained by evaporation of a hexanes/ethyl acetate (12:1) solution. Yield: 0.059 g, 16%. mp 84-85°C.

### S3. Refinement

Hydrogen atoms were placed at calculated positions and refined in the riding model approximation with distances of C–H = 0.95 and 0.99 Å for the benzene and methylene groups, respectively, and with *U_iso_*(H) = 1.2×*U_eq_*(C).

### Crystal data

**Table:**

C_13_H_10_N_2_O_4_	*Z* = 2
*M**_r_* = 258.23	*F*(000) = 268.00
Triclinic, *P*1	*D*_x_ = 1.474 Mg m^−^^3^
Hall symbol: -P 1	Mo *K*α radiation, λ = 0.71075 Å
*a* = 7.628 (3) Å	Cell parameters from 4826 reflections
*b* = 8.340 (3) Å	θ = 3.4–27.5°
*c* = 9.464 (4) Å	µ = 0.11 mm^−^^1^
α = 103.544 (8)°	*T* = 173 K
β = 92.555 (7)°	Prism, colorless
γ = 94.870 (7)°	0.17 × 0.15 × 0.10 mm
*V* = 582.0 (4) Å^3^	

### Data collection

**Table:**

Rigaku XtaLAB mini diffractometer	1866 reflections with *F*^2^ > 2σ(*F*^2^)
Detector resolution: 6.849 pixels mm^-1^	*R*_int_ = 0.038
ω scans	θ_max_ = 27.5°
Absorption correction: multi-scan (*REQAB*; Rigaku, 1998)	*h* = −9→9
*T*_min_ = 0.735, *T*_max_ = 0.989	*k* = −10→10
6052 measured reflections	*l* = −12→12
2648 independent reflections	

### Refinement

**Table:**

Refinement on *F*^2^	Secondary atom site location: difference Fourier map
*R*[*F*^2^ > 2σ(*F*^2^)] = 0.048	Hydrogen site location: inferred from neighbouring sites
*wR*(*F*^2^) = 0.114	H-atom parameters constrained
*S* = 1.02	*w* = 1/[σ^2^(*F*_o_^2^) + (0.0474*P*)^2^ + 0.1223*P*] where *P* = (*F*_o_^2^ + 2*F*_c_^2^)/3
2648 reflections	(Δ/σ)_max_ < 0.001
172 parameters	Δρ_max_ = 0.19 e Å^−^^3^
0 restraints	Δρ_min_ = −0.23 e Å^−^^3^
Primary atom site location: structure-invariant direct methods	

### Special details

**Table:**

Refinement. Refinement was performed using all reflections. The weighted *R*-factor (*wR*) and goodness of fit (*S*) are based on *F*^2^. *R*-factor (gt) are based on *F*. The threshold expression of *F*^2^ > 2.0 σ(*F*^2^) is used only for calculating *R*-factor (gt).

### Fractional atomic coordinates and isotropic or equivalent isotropic displacement parameters (Å^2^)

**Table:**

	*x*	*y*	*z*	*U*_iso_*/*U*_eq_	
O1	0.29405 (17)	0.79051 (18)	0.56711 (16)	0.0471 (4)	
O2	0.50674 (17)	0.97856 (16)	0.66006 (14)	0.0363 (4)	
O3	0.86890 (18)	1.24938 (16)	0.80274 (14)	0.0382 (4)	
O4	0.6835 (2)	1.29123 (18)	0.97055 (17)	0.0530 (5)	
N1	0.45029 (19)	0.84465 (19)	0.57775 (16)	0.0301 (4)	
N2	0.7777 (2)	1.20163 (18)	0.89199 (16)	0.0309 (4)	
C1	0.5742 (2)	0.7451 (2)	0.48873 (18)	0.0249 (4)	
C2	0.4972 (3)	0.6214 (3)	0.37138 (19)	0.0310 (4)	
C3	0.6030 (3)	0.5236 (3)	0.2806 (2)	0.0343 (5)	
C4	0.7836 (3)	0.5484 (3)	0.3085 (2)	0.0356 (5)	
C5	0.8591 (3)	0.6726 (3)	0.42590 (19)	0.0317 (4)	
C6	0.7571 (3)	0.7774 (2)	0.51854 (18)	0.0257 (4)	
C7	0.8516 (3)	0.9162 (3)	0.63788 (18)	0.0280 (4)	
C8	0.8178 (2)	0.9011 (2)	0.79215 (18)	0.0249 (4)	
C9	0.8194 (3)	0.7458 (3)	0.8234 (2)	0.0307 (4)	
C10	0.7870 (3)	0.7201 (3)	0.9604 (2)	0.0348 (5)	
C11	0.7497 (3)	0.8504 (3)	1.0715 (2)	0.0358 (5)	
C12	0.7464 (3)	1.0059 (3)	1.04560 (19)	0.0317 (4)	
C13	0.7820 (2)	1.0299 (2)	0.90776 (18)	0.0258 (4)	
H2	0.3726	0.6047	0.3541	0.0372*	
H3	0.5520	0.4400	0.1995	0.0412*	
H4	0.8570	0.4802	0.2471	0.0428*	
H5	0.9837	0.6865	0.4435	0.0381*	
H7A	0.8141	1.0231	0.6255	0.0336*	
H7B	0.9800	0.9184	0.6259	0.0336*	
H9	0.8434	0.6542	0.7483	0.0369*	
H10	0.7907	0.6126	0.9777	0.0418*	
H11	0.7265	0.8328	1.1648	0.0429*	
H12	0.7202	1.0964	1.1208	0.0380*	

### Atomic displacement parameters (Å^2^)

**Table:**

	*U*^11^	*U*^22^	*U*^33^	*U*^12^	*U*^13^	*U*^23^
O1	0.0241 (8)	0.0549 (10)	0.0606 (10)	0.0036 (7)	0.0085 (7)	0.0093 (8)
O2	0.0407 (8)	0.0371 (8)	0.0293 (7)	0.0118 (6)	0.0025 (6)	0.0016 (6)
O3	0.0466 (8)	0.0319 (7)	0.0369 (8)	−0.0007 (6)	0.0079 (7)	0.0107 (6)
O4	0.0715 (11)	0.0364 (8)	0.0522 (10)	0.0200 (8)	0.0250 (8)	0.0041 (7)
N1	0.0284 (9)	0.0365 (9)	0.0280 (9)	0.0084 (7)	0.0023 (7)	0.0115 (7)
N2	0.0344 (9)	0.0282 (8)	0.0279 (9)	0.0024 (7)	0.0002 (7)	0.0032 (7)
C1	0.0254 (9)	0.0278 (9)	0.0235 (9)	0.0059 (7)	0.0040 (7)	0.0085 (7)
C2	0.0279 (10)	0.0350 (10)	0.0298 (10)	−0.0004 (8)	−0.0030 (8)	0.0096 (8)
C3	0.0399 (11)	0.0310 (10)	0.0281 (10)	−0.0000 (8)	−0.0001 (8)	0.0008 (8)
C4	0.0409 (11)	0.0337 (10)	0.0314 (10)	0.0088 (8)	0.0078 (9)	0.0030 (8)
C5	0.0272 (10)	0.0388 (11)	0.0285 (10)	0.0045 (8)	0.0037 (8)	0.0059 (8)
C6	0.0282 (9)	0.0278 (9)	0.0220 (9)	0.0020 (7)	0.0027 (7)	0.0078 (7)
C7	0.0242 (9)	0.0291 (9)	0.0288 (10)	−0.0008 (7)	0.0032 (7)	0.0039 (8)
C8	0.0198 (9)	0.0276 (9)	0.0259 (9)	−0.0006 (7)	−0.0016 (7)	0.0052 (7)
C9	0.0283 (10)	0.0282 (10)	0.0331 (10)	0.0025 (8)	−0.0032 (8)	0.0031 (8)
C10	0.0366 (11)	0.0317 (10)	0.0374 (11)	−0.0006 (8)	−0.0042 (9)	0.0133 (9)
C11	0.0367 (11)	0.0430 (12)	0.0294 (10)	−0.0021 (9)	0.0002 (8)	0.0146 (9)
C12	0.0312 (10)	0.0371 (11)	0.0246 (10)	0.0004 (8)	0.0003 (8)	0.0043 (8)
C13	0.0227 (9)	0.0255 (9)	0.0281 (9)	−0.0001 (7)	−0.0010 (7)	0.0055 (7)

### Geometric parameters (Å, º)

**Table:**

O1—N1	1.229 (2)	C8—C13	1.397 (3)
O2—N1	1.2331 (18)	C9—C10	1.393 (3)
O3—N2	1.233 (3)	C10—C11	1.382 (3)
O4—N2	1.227 (3)	C11—C12	1.378 (3)
N1—C1	1.473 (3)	C12—C13	1.400 (3)
N2—C13	1.478 (3)	C2—H2	0.950
C1—C2	1.395 (3)	C3—H3	0.950
C1—C6	1.401 (3)	C4—H4	0.950
C2—C3	1.379 (3)	C5—H5	0.950
C3—C4	1.380 (3)	C7—H7A	0.990
C4—C5	1.394 (3)	C7—H7B	0.990
C5—C6	1.398 (3)	C9—H9	0.950
C6—C7	1.519 (3)	C10—H10	0.950
C7—C8	1.526 (3)	C11—H11	0.950
C8—C9	1.395 (3)	C12—H12	0.950
			
O1···C2	2.688 (3)	O3···H2^i^	2.8392
O1···C6	3.590 (3)	O3···H4^v^	3.0767
O2···O3	3.426 (2)	O3···H5^v^	2.7744
O2···O4	3.566 (2)	O3···H9^x^	3.5525
O2···N2	3.0953 (19)	O3···H10^x^	3.2126
O2···C2	3.532 (3)	O3···H10^vi^	3.2061
O2···C6	2.824 (3)	O3···H11^vi^	3.2348
O2···C7	2.732 (3)	O4···H2^i^	3.4020
O2···C8	2.825 (3)	O4···H3^vii^	2.5274
O2···C13	3.004 (3)	O4···H4^vii^	2.9142
O3···C7	2.840 (3)	O4···H10^x^	2.7197
O3···C8	2.875 (3)	O4···H11^iv^	3.3111
O3···C12	3.512 (3)	N1···H3^ii^	3.5241
O4···C8	3.554 (3)	N1···H7A^i^	3.1639
O4···C12	2.711 (3)	N1···H11^iv^	3.5821
N1···C7	3.070 (3)	N1···H12^iv^	3.1392
N1···C8	3.319 (3)	N2···H2^i^	3.3447
N2···C7	3.072 (3)	N2···H10^x^	3.3242
C1···C4	2.744 (3)	N2···H10^vi^	3.5553
C1···C8	3.280 (3)	C1···H11^xi^	3.5367
C2···C5	2.764 (3)	C2···H9^ii^	3.2897
C3···C6	2.836 (3)	C2···H11^xi^	3.3744
C5···C8	3.585 (3)	C3···H9^ii^	3.5691
C6···C9	2.979 (3)	C3···H10^xi^	3.4642
C8···C11	2.837 (3)	C3···H11^xi^	3.1208
C9···C12	2.759 (3)	C4···H9^xii^	3.4305
C10···C13	2.747 (3)	C4···H10^xi^	3.2996
O1···O2^i^	3.518 (3)	C4···H11^xi^	3.0494
O1···O3^i^	3.592 (3)	C5···H7A^v^	3.5358
O1···C3^ii^	3.403 (3)	C5···H11^xi^	3.2356
O1···C4^ii^	3.326 (3)	C6···H11^xi^	3.4859
O1···C5^iii^	3.492 (3)	C7···H7B^v^	3.3745
O2···O1^i^	3.518 (3)	C8···H12^vi^	3.5807
O2···O2^i^	3.131 (3)	C9···H2^ii^	3.2040
O2···N1^i^	2.981 (3)	C9···H3^ii^	3.0850
O2···C1^i^	3.060 (3)	C9···H4^xii^	3.2311
O2···C2^i^	3.421 (3)	C10···H3^ii^	2.9802
O2···C11^iv^	3.396 (3)	C11···H7B^vi^	3.5194
O2···C12^iv^	3.444 (3)	C12···H7B^vi^	3.5635
O3···O1^i^	3.592 (3)	H2···O2^i^	3.5542
O3···C2^i^	3.551 (3)	H2···O3^i^	2.8392
O3···C4^v^	3.362 (3)	H2···O4^i^	3.4020
O3···C5^v^	3.200 (3)	H2···N2^i^	3.3447
O3···C10^vi^	3.327 (3)	H2···C9^ii^	3.2040
O3···C11^vi^	3.338 (3)	H2···H5^iii^	3.2056
O4···C3^vii^	3.231 (3)	H2···H7A^i^	3.4920
O4···C4^vii^	3.421 (3)	H2···H9^ii^	2.5672
O4···C11^iv^	3.387 (3)	H2···H10^ii^	3.3613
N1···O2^i^	2.981 (3)	H3···O1^ii^	3.4775
N1···N1^i^	3.318 (3)	H3···O4^viii^	2.5274
N2···C10^vi^	3.492 (3)	H3···N1^ii^	3.5241
C1···O2^i^	3.060 (3)	H3···C9^ii^	3.0850
C2···O2^i^	3.421 (3)	H3···C10^ii^	2.9802
C2···O3^i^	3.551 (3)	H3···H9^ii^	3.1418
C2···C2^ii^	3.514 (3)	H3···H10^xi^	3.3329
C3···O1^ii^	3.403 (3)	H3···H10^ii^	2.9846
C3···O4^viii^	3.231 (3)	H3···H11^xi^	3.5234
C4···O1^ii^	3.326 (3)	H3···H12^viii^	3.1816
C4···O3^v^	3.362 (3)	H4···O1^ii^	3.3339
C4···O4^viii^	3.421 (3)	H4···O3^v^	3.0767
C5···O1^ix^	3.492 (3)	H4···O4^viii^	2.9142
C5···O3^v^	3.200 (3)	H4···C9^xii^	3.2311
C8···C12^vi^	3.544 (3)	H4···H9^xii^	2.6325
C10···O3^vi^	3.327 (3)	H4···H10^xi^	3.0462
C10···N2^vi^	3.492 (3)	H4···H10^xii^	3.5270
C11···O2^iv^	3.396 (3)	H4···H11^xi^	3.4275
C11···O3^vi^	3.338 (3)	H4···H12^viii^	3.2087
C11···O4^iv^	3.387 (3)	H5···O1^ix^	2.5794
C12···O2^iv^	3.444 (3)	H5···O3^v^	2.7744
C12···C8^vi^	3.544 (3)	H5···H2^ix^	3.2056
O1···H2	2.3718	H5···H5^xii^	3.5426
O2···H7A	2.3914	H5···H7A^v^	2.9778
O3···H7A	2.2068	H5···H7B^v^	3.4990
O3···H7B	3.0821	H5···H9^xii^	3.3980
O4···H12	2.4218	H7A···O1^i^	2.7985
N1···H2	2.5571	H7A···O2^i^	3.5612
N1···H7A	2.9988	H7A···N1^i^	3.1639
N2···H7A	2.6509	H7A···C5^v^	3.5358
N2···H7B	3.5330	H7A···H2^i^	3.4920
N2···H12	2.5617	H7A···H5^v^	2.9778
C1···H3	3.2585	H7A···H7B^v^	3.0193
C1···H5	3.2340	H7B···O1^ix^	2.7316
C1···H7A	2.8396	H7B···C7^v^	3.3745
C1···H7B	3.3700	H7B···C11^vi^	3.5194
C1···H9	3.3932	H7B···C12^vi^	3.5635
C2···H4	3.2392	H7B···H5^v^	3.4990
C3···H5	3.2571	H7B···H7A^v^	3.0193
C4···H2	3.2424	H7B···H7B^v^	3.0237
C5···H3	3.2674	H7B···H11^vi^	3.1837
C5···H7A	3.1423	H7B···H12^vi^	3.2696
C5···H7B	2.5180	H9···O3^xiii^	3.5525
C5···H9	3.0984	H9···C2^ii^	3.2897
C6···H2	3.3056	H9···C3^ii^	3.5691
C6···H4	3.2918	H9···C4^xii^	3.4305
C6···H9	2.6931	H9···H2^ii^	2.5672
C7···H5	2.6288	H9···H3^ii^	3.1418
C7···H9	2.6311	H9···H4^xii^	2.6325
C8···H10	3.2926	H9···H5^xii^	3.3980
C8···H12	3.3047	H10···O3^xiii^	3.2126
C9···H7A	3.3006	H10···O3^vi^	3.2061
C9···H7B	2.8685	H10···O4^xiii^	2.7197
C9···H11	3.2653	H10···N2^xiii^	3.3242
C10···H12	3.2441	H10···N2^vi^	3.5553
C11···H9	3.2542	H10···C3^xiv^	3.4642
C12···H10	3.2415	H10···C4^xiv^	3.2996
C13···H7A	2.6811	H10···H2^ii^	3.3613
C13···H7B	3.1139	H10···H3^xiv^	3.3329
C13···H9	3.2263	H10···H3^ii^	2.9846
C13···H11	3.2625	H10···H4^xiv^	3.0462
H2···H3	2.3363	H10···H4^xii^	3.5270
H3···H4	2.3305	H11···O1^iv^	3.5664
H4···H5	2.3258	H11···O2^iv^	2.8116
H5···H7A	3.3275	H11···O3^vi^	3.2348
H5···H7B	2.2737	H11···O4^iv^	3.3111
H5···H9	3.1830	H11···N1^iv^	3.5821
H7A···H9	3.5546	H11···C1^xiv^	3.5367
H7B···H9	2.8626	H11···C2^xiv^	3.3744
H9···H10	2.3236	H11···C3^xiv^	3.1208
H10···H11	2.3354	H11···C4^xiv^	3.0494
H11···H12	2.3352	H11···C5^xiv^	3.2356
O1···H3^ii^	3.4775	H11···C6^xiv^	3.4859
O1···H4^ii^	3.3339	H11···H3^xiv^	3.5234
O1···H5^iii^	2.5794	H11···H4^xiv^	3.4275
O1···H7A^i^	2.7985	H11···H7B^vi^	3.1837
O1···H7B^iii^	2.7316	H12···O1^iv^	2.8926
O1···H11^iv^	3.5664	H12···O2^iv^	2.9037
O1···H12^iv^	2.8926	H12···N1^iv^	3.1392
O2···H2^i^	3.5542	H12···C8^vi^	3.5807
O2···H7A^i^	3.5612	H12···H3^vii^	3.1816
O2···H11^iv^	2.8116	H12···H4^vii^	3.2087
O2···H12^iv^	2.9037	H12···H7B^vi^	3.2696
			
O1—N1—O2	122.50 (15)	N2—C13—C8	121.89 (16)
O1—N1—C1	118.50 (14)	N2—C13—C12	115.29 (14)
O2—N1—C1	119.01 (14)	C8—C13—C12	122.81 (17)
O3—N2—O4	122.91 (17)	C1—C2—H2	120.212
O3—N2—C13	119.29 (15)	C3—C2—H2	120.213
O4—N2—C13	117.80 (16)	C2—C3—H3	120.313
N1—C1—C2	115.46 (15)	C4—C3—H3	120.314
N1—C1—C6	121.63 (13)	C3—C4—H4	119.728
C2—C1—C6	122.88 (16)	C5—C4—H4	119.739
C1—C2—C3	119.57 (16)	C4—C5—H5	118.995
C2—C3—C4	119.37 (16)	C6—C5—H5	119.000
C3—C4—C5	120.53 (18)	C6—C7—H7A	108.677
C4—C5—C6	122.00 (17)	C6—C7—H7B	108.684
C1—C6—C5	115.58 (14)	C8—C7—H7A	108.672
C1—C6—C7	126.13 (15)	C8—C7—H7B	108.679
C5—C6—C7	118.27 (15)	H7A—C7—H7B	107.606
C6—C7—C8	114.32 (15)	C8—C9—H9	118.783
C7—C8—C9	118.53 (15)	C10—C9—H9	118.770
C7—C8—C13	125.89 (17)	C9—C10—H10	119.901
C9—C8—C13	115.57 (17)	C11—C10—H10	119.902
C8—C9—C10	122.45 (16)	C10—C11—H11	120.303
C9—C10—C11	120.2 (2)	C12—C11—H11	120.312
C10—C11—C12	119.39 (19)	C11—C12—H12	120.215
C11—C12—C13	119.57 (16)	C13—C12—H12	120.219
			
O1—N1—C1—C2	17.2 (3)	C4—C5—C6—C1	2.3 (3)
O1—N1—C1—C6	−164.73 (16)	C4—C5—C6—C7	−176.20 (17)
O2—N1—C1—C2	−162.89 (15)	C1—C6—C7—C8	65.5 (3)
O2—N1—C1—C6	15.2 (3)	C5—C6—C7—C8	−116.10 (18)
O3—N2—C13—C8	−28.6 (2)	C6—C7—C8—C9	42.9 (2)
O3—N2—C13—C12	151.93 (13)	C6—C7—C8—C13	−136.09 (15)
O4—N2—C13—C8	152.16 (14)	C7—C8—C9—C10	−178.93 (13)
O4—N2—C13—C12	−27.3 (2)	C7—C8—C13—N2	−1.7 (3)
N1—C1—C2—C3	178.99 (15)	C7—C8—C13—C12	177.81 (13)
N1—C1—C6—C5	179.61 (15)	C9—C8—C13—N2	179.33 (13)
N1—C1—C6—C7	−2.0 (3)	C9—C8—C13—C12	−1.2 (3)
C2—C1—C6—C5	−2.5 (3)	C13—C8—C9—C10	0.2 (3)
C2—C1—C6—C7	175.89 (17)	C8—C9—C10—C11	0.7 (3)
C6—C1—C2—C3	1.0 (3)	C9—C10—C11—C12	−0.6 (3)
C1—C2—C3—C4	0.8 (3)	C10—C11—C12—C13	−0.4 (3)
C2—C3—C4—C5	−1.0 (3)	C11—C12—C13—N2	−179.15 (14)
C3—C4—C5—C6	−0.7 (3)	C11—C12—C13—C8	1.4 (3)

Symmetry codes: (i) −*x*+1, −*y*+2, −*z*+1; (ii) −*x*+1, −*y*+1, −*z*+1; (iii) *x*−1, *y*, *z*; (iv) −*x*+1, −*y*+2, −*z*+2; (v) −*x*+2, −*y*+2, −*z*+1; (vi) −*x*+2, −*y*+2, −*z*+2; (vii) *x*, *y*+1, *z*+1; (viii) *x*, *y*−1, *z*−1; (ix) *x*+1, *y*, *z*; (x) *x*, *y*+1, *z*; (xi) *x*, *y*, *z*−1; (xii) −*x*+2, −*y*+1, −*z*+1; (xiii) *x*, *y*−1, *z*; (xiv) *x*, *y*, *z*+1.
